# Temperature Affects the Biological Control of Dinoflagellates by the Generalist Parasitoid *Parvilucifera rostrata*

**DOI:** 10.3390/microorganisms10020385

**Published:** 2022-02-07

**Authors:** Matthew Schmitt, Aaron Telusma, Estelle Bigeard, Laure Guillou, Catharina Alves-de-Souza

**Affiliations:** 1 Marine Sciences, University of North Carolina Wilmington, Wilmington, NC 28409, USA; matte.schmitt@gmail.com; 2 Algal Resources Collection, Center for Marine Sciences, University of North Carolina Wilmington, Wilmington, NC 28409, USA; aarontelusma@gmail.com; 3 CNRS & Sorbonne Université, Station Biologique de Roscoff, Place Georges Teissier, CS90074, 29688 Roscoff, France; estelle.bigeard@sb-roscoff.fr (E.B.); lguillou@sb-roscoff.fr (L.G.)

**Keywords:** parasitic control, dinoflagellate blooms, functional response, temperature effect

## Abstract

The increase in emerging harmful algal blooms in the last decades has led to an extensive concern in understanding the mechanisms behind these events. In this paper, we assessed the growth of two blooming dinoflagellates (*Alexandrium minutum* and *Heterocapsa triquetra*) and their susceptibility to infection by the generalist parasitoid *Parvilucifera rostrata* under a temperature gradient. The growth of the two dinoflagellates differed across a range of temperatures representative of the Penzé Estuary (13 to 22 °C) in early summer. *A. minutum* growth increased across this range and was the highest at 19 and 22 °C, whereas *H. triquetra* growth was maximal at intermediate temperatures (15–18 °C). Interestingly, the effect of temperature on the parasitoid infectivity changed depending on which host dinoflagellate was infected with the dinoflagellate responses to temperature following a positive trend in *A. minutum* (higher infections at 20–22 °C) and a unimodal trend in *H. triquetra* (higher infections at 18 °C). Low temperatures negatively affected parasitoid infections in both hosts (i.e., “thermal refuge”). These results demonstrate how temperature shifts may not only affect bloom development in microalgal species but also their control by parasitoids.

## 1. Introduction

Unicellular eukaryotic parasitoids play essential roles in phytoplankton bloom dynamics but they are often overlooked [1]. These organisms are of special interest as the controlling agents of dinoflagellate species and several of them cause harmful algal blooms (HABs) [2]. The biological control of dinoflagellate blooms by unicellular eukaryote parasitoids has been shown on a few occasions [3,4,5,6], with their effect being suggested to be similar to grazing [7]. Several parasitoid species that infect dinoflagellates may also enter dormancy in the sediment and emerge when conditions are more favorable [8]. The ability to switch hosts, putatively easiest in generalistic parasitoids, is key in the control of invasive HAB species [6]. However, little is known about the potential of generalist parasitoids in controlling dinoflagellate bloom dynamics.

Species of the genus *Parvilucifera* have been reported to infect a broad range of dinoflagellate hosts, mostly in culture conditions [4], although a host preference has been suggested to occur in natural assemblages [9,10]. These generalist parasitoids have a life cycle starting with nanoflagellate infective stages (i.e., zoospores) that invade dinoflagellate host cells, which lose motility and sink [4]. The primary intracellular stage of infection (i.e., the trophocyte) is apparent after ~24 hours [11]. After 3–4 days, the trophocyte matures to a sporocyte, which turns from white to dark when the zoospores are mature. Hundreds of zoospores per sporocyte are released, specifically in the presence of fresh dinoflagellate hosts [12], ready to restart a new infective cycle [4].

Field studies usually focus on describing the *Parvilucifera* host range and host–parasitoid dynamics [7,9,13] with limited information on the environmental conditions in which the parasitoid is present with respect to the hosts. However, understanding the impacts of abiotic factors that can influence *Parvilucifera* infectivity is crucial for determining the efficiency of these organisms to terminate dinoflagellate blooms. A few of these factors have been previously examined for *Parvilucifera sinerae*, such as the effect of turbulence on the infection rates [14] and temperature on the parasitoid generation time (i.e., time for the completion of infection and release of zoospores) [7]. However, the effect of temperature on parasitoid infectivity is yet to be determined. A re-examination of temperature is required to understand these parasitoids due to the strong influence of this variable on the host growth rates, thereby affecting the bloom dynamics and succession patterns observed in the spring–summer transition. 

Parasitoids are, in essence, specialized predators that live in a close association with their prey (the hosts), ultimately leading to their death [4]. As such, their infection rates are determined by functional responses (i.e., changes in the infection rates of hosts with host density [15]). Eukaryotic parasitoids infecting phytoplankton follow a Type II functional response e.g., [2,11], which is characterized by an increase in the infection rate as the host:zoospore ratio increases followed by a deceleration until a maximal infection rate is reached after a certain host abundance is achieved [15]. This is because the time searching (“attack rate”) for hosts decreases as the host:zoospore ratios increase, but the time needed to complete the infection (“handling time”) remains the same. The estimation of these two parameters in determining the parasitoid functional response is key for understanding the effect of abiotic factors on parasitoid–host dynamics.

In this paper, we assessed how the effect of temperature on the functional response of the generalist parasitoid *Parvilucifera rostrata* mediates the role of parasitism on the coexistence of their dinoflagellate hosts. For that, we based our experiments on the temperature range (13 °C to 22 °C) observed during dinoflagellate blooms at the Penzé Estuary (France), where *P. rostrata* was observed infecting both *Alexandrium minutum* and *Heterocapsa triquetra* (the predominant blooming dinoflagellates in this system during early summer). Strains from these species were isolated from sympatric populations and used in experiments to assess the effect of temperature on both the host growth and the parameters determining the parasitoid functional response (i.e., the attack rate and handling time) as well as other parasitoid attributes affecting the infection rates (i.e., zoospores produced per infected host and zoospore mortality). The metrics from the experimental data were included in numerical simulations to assess the role of temperature and parasitism in the bloom dynamics of the two dinoflagellate species.

## 2. Methods

### 2.1. Origin of the Strains and Culture Conditions

Host and parasite strains were isolated from the Penzé Estuary (northwest of France, English Channel; 48°37′ N, 3°56′ W). Cultures of *Alexandrium minutum* (Paralex 176/RCC3018) and *Heterocapsa triquetra* (HT150/RCC4398) were obtained by microcapillary single-cell isolation [16] and maintained in an L1 medium (salinity 27) supplemented with 2% (*v*/*v*) soil extract [17] at 20 °C with a 14:10 h light:dark cycle and 60 μE m^−2^ s^−1^ irradiance. Clonal *Parvilucifera rostrata* strains (RCC2800, RCC2823, and RCC2982) were isolated using *A. minutum* (Paralex 176/RCC3018) as the primary host as described in Lepelletier et al. [18] and kept by successive transfers to exponentially grow the uninfected host cultures once a week. For all the experiments described below, the *P. rostrata* cultures were previously acclimated to the hosts and experimental conditions for at least two months.

### 2.2. Parasitoid–Host Dynamics

Infections of *P. rostrata* RCC2823 on *A. minutum* and *H. triquetra* were followed at 20 °C over one week to obtain an estimate of the time required by *P. rostrata* to complete each infective stage. The dinoflagellate hosts (final concentration of 6000 cells mL^−1^) and freshly released zoospores were mixed in 50 mL flasks using an initial zoospore:host ratio of 50:1 (the steps for harvesting the fresh zoospores are illustrated in Figure S1). Samples were obtained daily after inoculation and fixed with glutaraldehyde (1%). Each stage of infection was quantified using a Sedgewick Rafter chamber. Glutaraldehyde was selected over Lugol’s solution because the latter changes the coloration of the cells, making it difficult to distinguish between the different parasite infective stages.

For the determination of parasitoid generation times on both the hosts at different temperatures (13 °C, 15 °C, 18 °C, 20 °C, and 22 °C), cultures of *A. minutum* and *H. triquetra* were inoculated with freshly harvested zoospores from *P. rostrata* RCC28223 at a high zoospore:host ratio (over 50:1) and observed once a day using light microscopy to determine when each stage of parasitoid intracellular and extracellular development was first observed (from early trophocyte to zoospore release). 

### 2.3. Zoospore Viability

The effect of the zoospore age on parasitoid infectivity was assessed under three temperatures (13 °C, 18 °C, and 22 °C). The dinoflagellate hosts (2000 cells mL^−1^) and dense freshly released zoospores (>100,000 cells mL^−1^) from the strain *P. rostrata* RCC28223 were kept separately in 50 mL tissue culture flasks. Every two hours, a 1 mL aliquot from both the hosts and zoospores were added to 2-mL wells of a 24-well plate in triplicate (final host concentration of 1000 cells mL^−1^ and a zoospore:host ratio higher than 50:1). After 10 h, inoculations were performed every hour until the zoospores were no longer motile in the zoospore inoculum (after 20 h). Control wells with only the hosts were also included. After two (18 °C and 22 °C) or three days (13 °C), the well-plate contents were fixed with glutaraldehyde (1%) and stored at 4 °C until quantification using an inverted microscope. The data were fitted to a four-parameter logistic model using the *nlsLM* function in the ‘minpack.lm’ package in R, based on the equation:

(1) $$y=a+\frac{d}{1+e^{b\left(x-c\right)}}$$

where *a* and *d* are the *y*-intersects for the maximum and minimum infections (i.e., upper and lower asymptotes), respectively, *b* is the slope of the curve, and *c* is the *x* value where the middle point between *a* and *d* is observed. Here, we used *c* to depict the zoospore age (in hours) when its infectivity was reduced by 50%.

Due to the difficulty in estimating the zoospore survival, the decay in the infectivity of the zoospores with age was used as a proxy to estimate the zoospore viability based on a two-parameter exponential decay model as described in Coats and Park [2]. A comparison of the zoospore mortality among the different temperatures was performed by Kruskal–Wallis tests using the *kruskal.test* function in the basic ‘stats’ package in R followed by a post-hoc Dunn test using the *dunnTest* function in the ‘FSA’ package [19]. 

### 2.4. Zoospore Yield

Fifty late sporocytes were measured for both species at each temperature. A comparison of the late zoospore diameter among the different temperatures was performed by a Kruskal–Wallis test. Given the lack of significance (see results), the data from the inspection of the sporocytes from the 20 °C incubations were used to establish a relationship between the sporocyte size and zoospore production. For both hosts, late sporocytes were individually isolated (*n* = 50), placed on a glass slide, and gently covered with a glass coverslip whilst observing them under a microscope at 40×. The sporocytes were photomicrographed and then physically burst by applying gentle but targeted pressure over the coverslip to make them release the zoospores. Photomicrographs of the process were obtained to quantify the number of released zoospores per host. These data were fittedthrough a linear regression using the function *lm* in the basic ‘stats’ package in R software [20]. The late sporocyte diameter and zoospore production in both hosts were compared by Mann–Whitney tests using the *wilcox.test* function in the basic ‘stats’ package in R. 

### 2.5. Host Growth and Parasitoid Functional Response

The growth of the dinoflagellate hosts *A. minutum* and *H. triquetra* was assessed at five temperatures (13 °C, 15 °C, 18 °C, 20 °C, and 22 °C) in three replicates. The cultures were inoculated in 50 mL of a fresh L1 medium supplemented with a 2% soil extract at an initial concentration of 500 cells mL^−1^. Samples (1 mL) were obtained daily and fixed with Lugol’s solution and quantified in a Sedgewick Rafter chamber. The growth rates were estimated assuming exponential growth [21]:

(2) $$\mu =\frac{1}{t_{2}-t_{1}}ln\frac{N_{2}\;}{N_{1}}$$

where *μ* is the growth rate (d^−1^) and *N*_1_ and *N*_2_ are the dinoflagellate abundances at previous (*t*_1_) and consecutive days (*t*_2_), respectively. The growth rates of the two hosts in each temperature were compared by Mann–Whitney tests. 

The effect of the five mentioned temperatures on the functional response of the three *P. rostrata* strains (RCC2800, RC2823, and RCC2982) infecting both *A. minutum* and *H. triquetra* was assessed by determining the infection rates under increasing zoospore:host ratios (1:1, 2:1, 3.5:1, 5:1, 10:1, 20:1, 50:1, and 100:1) in three replicates using 24-well culture plates (2-mL wells). Dinoflagellates without parasites were also included as negative controls to estimate the percentage of increase in the hosts due to growth and discounted from uninfected cells in the infected treatments to avoid underestimating the infection percentage. After the period of time at which late trophocytes were expected to be observed (three days for 13–15 °C and two days for 18–22 °C), glutaraldehyde (1%) was added to all wells and the plates were stored at 4 °C until quantification. The late trophocyte stage was chosen because it is easily recognizable and is more reliable than sporocytes (i.e., longer viability and no disappearance due to zoospore release). The incubation time difference between 13–15 °C and 18–22 °C did not affect the maximal infected abundances as all infections were expected to occur within 15 h after inoculation due to the limited short-term viability of the *P. rostrata* zoospores (see results). The infected and uninfected dinoflagellates were counted directly in the 24-well plates with an inverted microscope after the plates achieved room temperature and the content of each well was gently homogenized by pipetting. Counts were performed in a transect across the center of each well at 20 × magnification to determine the percentage of infected cells. 

The parasite functional response was estimated based on the Holling II equation [15]:

(3) $$y=\frac{Tax}{1+abx}$$

where *y* is the infection rate (%), *T* is the total time available for the parasite to search for a host, *x* is the zoospore:host ratio, *a* (the slope of the curve) is the instantaneous rate of discovery (i.e., the attack rate), and *b* is the time for the parasite to infect the host. Here, the attack rate was estimated by fitting the infection rates against the zoospore:host ratios using a non-linear least square model based on Equation (3) [15]:

(4) $$y=\frac{ax}{1+bx}$$

and subsequently converted to daily rates:

(5) $$a^{\prime }\;\left(d^{-1}\right)=\frac{a}{100\%}\times \frac{24\;\left(h/\mathrm{day}\right)}{T\left(h\right)}$$

where *a*′ is the attack rate given in a percentage (and, therefore, divided by 100). *T* was established as 15 h because this was the maximum period of time after the zoospore release when they could establish infections (see results).

For each *P. rostrata* strain, the differences in the parasite infectivity (percentage of infection at 50:1) and search rates among the different temperatures were tested by Kruskal–Wallis tests followed by a post-hoc Dunn test. We selected the 50:1 ratio as it corresponded with the inflection point in the fit curves.

### 2.6. Modeling Approach

We used numerical simulations to access how temperature mediates the effect of infections by *P. rostrata* on the competitive interactions between *A. minutum* and *H*. *triquetra*. The model was based on differential equations modified from Salomon and Stolte [22] and used the parameters related to the parasitoid–host dynamics obtained in this study (Table 1). The average values of the three *P. rostrata* strains were used to model the parasitoids. Changes in the two host concentrations were simulated with the two equations below (subscripts 1 and 2 are used to indicate the parameters and variables related to *A. minutum* and *H*. *triquetra*, respectively):

(6) $$\frac{dH_{1}}{dt}=r_{1}H_{1}\frac{\left(K-\left(H_{1}+H_{2}\right)\right)}{K}-\frac{a_{1}H_{1}}{1+a_{1}h_{1}H_{1}}P$$

(7) $$\frac{dH_{2}}{dt}=r_{2}H_{2}\frac{\left(K-\left(H_{1}+H_{2}\right)\right)}{K}-\frac{a_{2}H_{2}}{1+a_{2}h_{2}H_{2}}P$$

where *H* represents the uninfected hosts and *r* is the host maximal growth rate. *K* is the carrying capacity, and it was defined here as 45,000 cells mL^−1^, the maximum cell concentration observed in the *A. minutum* cultures.

Changes in the parasite stages were estimated based on the equations:

(8) $$\frac{dP}{dt}=\varepsilon_{1}\frac{I_{1}}{h_{1}}+\varepsilon_{2}\frac{I_{2}}{h_{2}}-\frac{a_{1}H_{1}}{1+a_{1}h_{1}H_{1}}P-\frac{a_{2}H_{2}}{1+a_{2}h_{2}H_{2}}P-mP$$

(9) $$\frac{dI_{1}}{dt}=\frac{aH_{1}}{1+ah_{1}H_{1}}P-\frac{I_{1}}{h_{1}}$$

(10) $$\frac{dI_{2}}{dt}=\frac{a_{2}H_{2}}{1+a_{2}h_{2}H_{2}}P-\frac{I_{2}}{h_{2}}$$

where *I* represents the infected hosts, *P* is the zoospore abundance, *a* is the search rate, *h* (i.e., parasite generation time) is the handling time, *ε* is the number of zoospores released per infected host, and *m* is the zoospore mortality rate. Although *h* could be potentially estimated from Equation (4) [15], in our case, it expressed the time required by the zoospores to infect the hosts because the experiments for the parasite numerical response only included one infective cycle. As the handling time referred to the time needed for the parasitoid to go from one host to the next one, we considered the period of time from infection to zoospore release to be a better approximation for *h*, as considered in previous models accessing parasite infections in dinoflagellates [3,22].

## 3. Results and Discussion

### 3.1. Parasitoid–Host Dynamics

**Figure 1:**
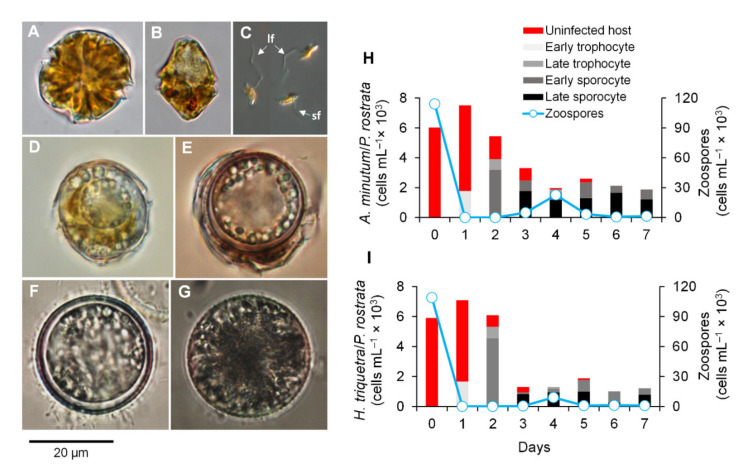
Dinoflagellate hosts *Alexandrium minutum* (**A**) and *Heterocapsa triquetra* (**B**). Parvilucifera rostrata stages from infections in *A. minutum*: zoospores (**C**) (lf = long flagellum, sf = short flagellum), early trophocyte (**D**), late trophocyte (**E**), early sporocyte (**F**), and late sporocyte (**G**); Parasitoid–host dynamics of *P. rostrata* RCC2823 infecting *A. minutum* (**H**) and *H. triquetra* (**I**) at 20 °C. All photomicrographs (**A**–**G**) are from living cells, except for zoospores (**C**) that were fixed with Lugol’s solution.

The infection dynamics of *P. rostrata* RCC2823 at 20 °C over a 7-day period were followed by inoculating the dinoflagellate hosts *A. minutum* (Figure 1A) and *H. triquetra* (Figure 1B) with freshly harvested zoospores (Figure 1C) at an initial zoospore:host ratio of 50:1. The morphology of all parasitoid stages (Figure 1D–G) was similar to the one reported in the original description of *P. rostrata* [18]. Similar infective patterns were observed for both hosts (Figure 1H,I). Early trophocytes were observed predominantly on Day 1 and both late trophocytes and early sporocytes were detected on Day 2. Late sporocytes were first observed on Day 3 followed by a peak in zoospore release on Day 4. These infection dynamics and generation times were similar to those reported from *P. sinerae* at 20 °C [11]. Late sporocytes were then the predominant parasite stage after Day 5 with no other noticeable zoospore release. After this point, zoospores were only released following the addition of fresh hosts or filtrate from host cultures (data not shown). This agreed with previous observations for *Parvilucifera sinerae*, suggesting that late sporocytes acted both as virulent and dormant stages activated by the algal metabolite dimethylsulphide (DMS) that served as a density-dependent cue of the presence of potential hosts [12]. Although the infective dynamics were similar in both hosts, the abundance of *P. rostrata* infective stages and zoospores were consistently lower in *H. triquetra*, potentially due to cells being lost instead of transformed into infective stages. 

**Figure 2:**
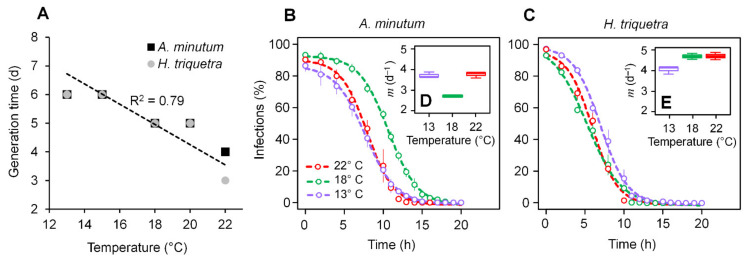
Effect of temperature on *Parvilucifera rostrata* RCC2823 generation time from infections in the dinoflagellates *Alexandrium minutum* and *Heterocapsa triquetra* (**A**); decrease in zoospore infectivity with age in *P. rostrata* RCC2823 infecting *A. minutum* (**B**) and *H. triquetra* (**C**). The effect of temperature on the mortality of *P. rostrata* RCC2823 zoospores produced from infections in *A. minutum* (**D**) and *H. triquetra* (**E**) are shown as insets in (**B**) and (**C**), respectively.

An inversely proportional relationship was observed between temperature and the *P. rostrata* generation time in both hosts, from five days at 13 °C to three or four days at 22 °C (Figure 2A). Zoospore release in both hosts was observed at the same time at 13–15 °C 267 (6 days) and 18–20 °C (5 days). At 22 °C, zoospores were detected at Day 3 and 4 for in- 268 fections on *H. triquetra* and *A. minutum*, respectively. These results agreed with previous observations, suggesting that colder temperatures resulted in longer generation times for *P. sinerae* [7].

### 3.2. Zoospore Infectivity and Viability

A decrease in the zoospore infectivity of *P. rostrata* RCC2823 with age showed an inverse sigmoid response in both hosts (Figure 2B,C) (R^2^ = 0.87–0.92 for *A. minutum* and R^2^ = 0.85–0.89 for *H. triquetra*; *p* < 0.05). The zoospores showed maximum infectivity within 4–5 h of their release from the late sporocytes, with infective rates declining to zero after 12 h in *H. triquetra* and 13–15 h in *A. minutum*. Zoospore viability in the infections of *A. minutum* was optimal at 18 °C with an inverse unimodal distribution (Figure 2B). In *H. triquetra*, the 50% decrease in zoospore infectivity took slightly longer at 13 °C (7.03 ± 0.36 h) when compared with those observed at 18 °C and 22 °C (5.31 ± 0.04 h and 8.93 ± 0.46 h, respectively) (Figure 2C). Although the mechanism behind these differences in temperature optima for parasitoid viability per host was unclear, it could be related to the physiological attributes of the host (e.g., allelopathic potential of the respective dinoflagellate hosts across this temperature range). Zoospore mortality rates were estimated using an exponential decay regression (Figure 2D,E) with the lowest (2.7 ± 0.11 d^−1^) and highest (4.68 ± 0.68 d^−1^) zoospore mortalities observed in *A. minutum* and *H. triquetra*, respectively, at 18 °C. However, these differences in both zoospore viability and mortality across the three temperatures were not statistically significant (Kruskal–Wallis tests; *p* > 0.05).

Ours constitutes the first comprehensive examination of the decrease of *Parvilucifera* infectivity with zoospore age. In a preliminary experiment, it was observed that *Parvilucifera* sp. zoospores were not able to start a novel infection 7 h after their release, which led to an estimated zoospore mortality rate of 2.5 d^−1^ [23]. Although this value was comparable with the lower zoospore mortalities observed here from the infections in *A. minutum (*~2.7 d^−1^) at 20 °C, it constituted an underestimation of the higher mortalities observed in the zoospores from the infections in *A. minutum* (~3.8 d^−1^) and *H. triquetra (*~4.7 d^−1^) at 18 °C. It is notable that the zoospore mortality of *Parvilucifera* was considerably higher than that observed for *Amoebophrya* (0.25–1.02 d^−1^) [2], another parasitoid genus infecting dinoflagellates that showed more restricted host ranges. This is particularly relevant as one of the factors explaining the coexistence of both parasitoid genera in the Penzé Estuary sharing the same hosts, as the putative advantage of *Parvilucifera* infecting several hosts could be compensated by lower zoospore mortalities in the more specialized *Amoebophrya.*

### 3.3. Zoospore Yield

**Figure 3:**
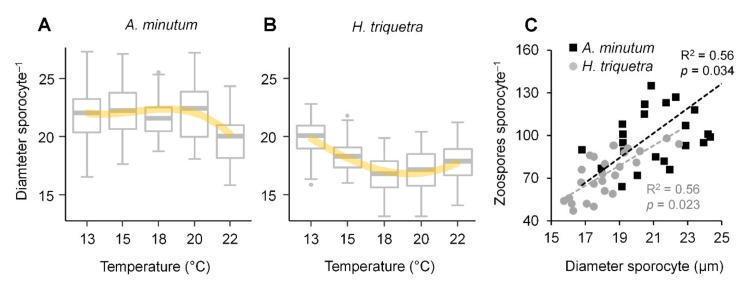
Effect of temperature on late sporocyte diameter in *Parvilucifera rostrata* RCC2823 infecting the dinoflagellates *Alexandrium minimum* (**A**) and *Heterocapsa triquetra* (**B**). Positive relationship between late sporocyte diameter and zoospores produced per infection in *Parvilucifera rostrata* RCC2823 infecting *A. minutum* and *H. triquetra* (**C**).

The sporocyte diameter in *A. minutum* was similar in all tested temperatures (Figure 3A), whereas a slight trend of a decreasing sporocyte diameter with an increasing temperature (although not statistically significant; *p* > 0.05) was observed in *H. triquetra* (Figure 3B). A positive interaction was observed between the diameter of the late sporocytes and the number of zoospores released per infection (Figure 3C). This agreed with previous studies for *Parvilucifera infectans* [24] and *P. sinerae* [11], which reported zoospore production to be related to the host size and late sporocyte diameter. The zoospore yield varied from 64 to 241 (average: 105 ± 34) in *A. minutum* and 47 to 98 (average: 73 ± 16) in *H. triquetra.* Although the late sporocyte diameter and the number of zoospores released per infection were slightly higher in *A. minutum* when compared with *H. triquetra*, this difference was not statistically significant (*p* > 0.05). The zoospore production per infected host in *P. rostrata* RCC2823 infecting *A. minutum* was much lower than reported for *P. sinerae* infecting the same dinoflagellate species (200–300 zoospores per infection) [11].

### 3.4. Host Growth and Parasitoid Functional Response

**Figure 4:**
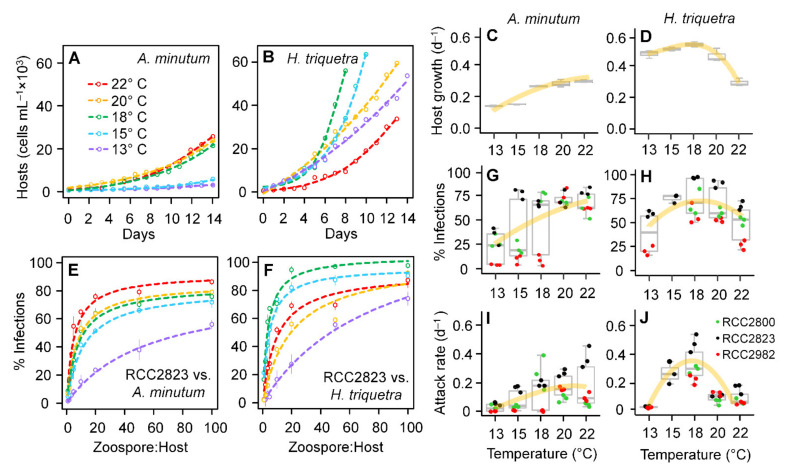
Effect of temperature on cell density (**A**,**B**) and growth rates (**C**,**D**) of the dinoflagellate hosts *Alexandrium minutum* (**A**,**C**) and *Heterocapsa triquetra* (**B**,**D**) in the absence of parasitoids; effect of temperature on the functional response of *Parvilucifera rostrata* RCC2823 on both *A. minutum* (**E**) and *H. triquetra* (**F**) (functional responses of *P. rostrata* RCC2800 and RCC2982 on both hosts are shown in Figure S2); infection rates (**G**,**H**) and attack rates (**I**,**J**) for the three *Parvilucifera* strains infecting the two hosts.

The two dinoflagellate hosts showed different patterns in their growth in the absence of the parasitoid under the tested temperature gradient over a 14-day period (Figure 4A–D). In *A. minutum*, the maximum cell concentrations (~20,000 cells mL^−1^; Figure 4A) and growth rates (0.26 ± 0.2 d^−1^; Figure 4C) were observed at 18–22 °C whereas reduced abundances (~6,000 cells mL^−1^) and growth (0.16 ± 0.1 d^−1^) were associated with lower temperatures (13–15 °C). An opposite pattern was observed in *H. triquetra* with higher cell concentrations (~56,000 cells mL^−1^; Figure 2B) and growth rates (0.51 ± 0.02 d^−1^; Figure 4D) observed from 13 °C to 20 °C (maximal values at 18 °C). 

Similar patterns were observed in the effect of temperature on the functional responses of *P. rostrata* (Figure 4E,F and Figure S2) with a positive effect on *A. minutum* infections (Figure 4G) and a unimodal response on *H. triquetra* (Figure 4H). The three *P. rostrata* strains (RCC2800, RCC2823, and RCC2982) infecting *A. minutum* showed lower infective rates at 13 °C (medians: 24–34%), whereas higher infectivity was observed at 20–22 °C (medians: 69–79%). However, the responses at intermediate levels were slightly different among the three strains at intermediate temperatures (15–18 °C). The Kruskal–Wallis test resulted in significant differences between the five temperatures (*p* < 0.01), whereas the post-hoc Dunn test indicated that this result was related to the differences only between 13 °C and 20 °C (*p* < 0.01) and between 13 °C and 22 °C (*p* < 0.01). In *H. triquetra*, no difference was observed among the *P. rostrata* strains as the three of them showed lower infectivity rates at 13 °C (median: 43%) and 22 °C (median: 53%) and higher infectivity at 15–18 °C (medians: 69–89%) (Figure 4H; Kruskal–Wallis test, *p* < 0.05). This pattern was confirmed by a pairwise comparison using post-hoc Dunn tests (*p* > 0.05). The attack rates estimated from the slope of the regression curves based on Equation (4) (Figure 4I,J) were significantly different among the tested temperatures for both strains (Kruskal–Wallis test; *p* < 0.01). The patterns of the parasite attack rates related to temperature in *A. minutum* were similar to those detected for the infection rates in the three *P. rostrata* strains. However, the effect of temperature on the attack rates of *P. rostrata* infecting *H. triquetra* (Figure 4J) showed a slightly different pattern than that observed for the infection rates (Figure 4H) with the attack rates at 13 °C, 20 °C, and 22 °C being considerably lower than those observed at 15 °C and 18 °C (post-hoc Dunn tests; *p* < 0.05).

These results suggested that the effect of temperature on the parasitoid infectivity was ultimately defined by the interaction of the individual responses on the host growth rates and parasitoid infective parameters (i.e., the attack rate and handling time). In *A. minutum*, the positive linear response between temperature and the *P. rostrata* infections (Figure 4G) was congruent with both the host growth rates and parasitoid parameters, showing lower values at 13–15 °C and higher values at 28–22 °C. On the other hand, the unimodal response of temperature on the *H. triquetra* infections (Figure 4F) was associated with opposing trends for the host growth rates and parasitoid parameters with low temperatures having a positive effect on the host growth and a negative effect on the parasitoid parameters, allowing the host to escape parasitoid control. This “thermal refuge” for host growth at low temperatures has been previously suggested in field surveys investigating the relevance of chytrid infections for the population dynamics of freshwater cyanobacteria and natural marine diatoms infected by the nanoflagellate *Cryothecomonas aestivalis*, and could be a common mechanism shaping the parasitoid–host dynamics in plankton assemblages.

### 3.5. Numerical Simulations

**Figure 5:**
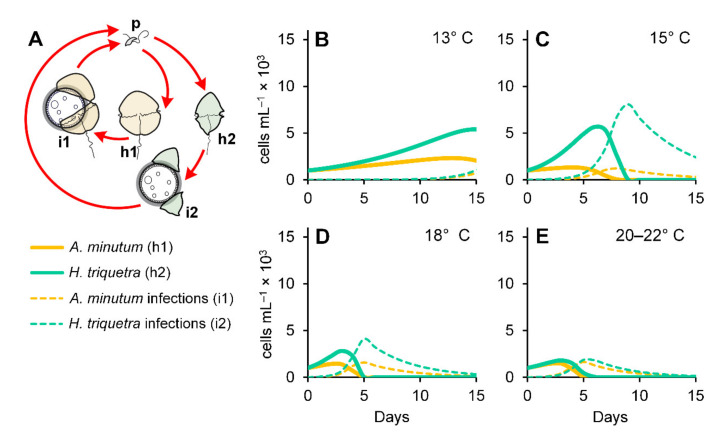
Conceptual model (**A**) used for numerical simulations of *Parvilucifera* infections in the dinoflagellates *Alexandrium minutum* and *Heterocapsa triquetra* under different temperatures (**B**–**E**). *p* = *P. rostrata* zoospores; h1 *=* uninfected *A. minutum*; h2 *=* uninfected *H. triquetra*; i1 = *A*. *minutum* infections; i2 = *H. triquetra* infections.

We used numerical simulations to assess how the differential effect of temperature on *P. rostrata* infectivity potentially affected the bloom dynamics of their dinoflagellate hosts (Figure 5A). The simulations of the hosts growing by themselves and without the parasitoid (Figure S3A,B) generated trends similar to those observed in the experiments (Figure 4A,B) with the highest *H. triquetra* and *A. minutum* abundances obtained at 18 °C and 18–22 °C, respectively. The simulations of the two dinoflagellates growing together resulted in a decrease of the *A. minutum* maximum abundances from 15–20 °C with the most pronounced decrease at 18 °C (Figure S3C). Incidentally, 18 °C is the optimal temperature for *H. triquetra*, which was only slightly affected by the presence of *A. minutum* at all temperatures (Figure S3D). A parasitoid addition to the simulations with both hosts growing by themselves (Figure S4) and together (Figure 5B–E) resulted in an expected pronounced decline in the maximal dinoflagellate abundances (about one-third of what was obtained in the simulations without parasites). However, this decline was more pronounced when both hosts were simulated together. This was explained by an increase in infectivity due to the availability of more hosts determined by the parasitoid functional response. Changes in the carrying capacity (*K*) affected the maximal abundances of the dinoflagellate hosts and parasitoids but did not affect the observed dynamics (data not shown). Shifts in host temperature optima were determined by parasitoid infections with maximal abundances observed at 13–15 °C for both dinoflagellates. This outcome was congruent with previous field mesocosm surveys where warming (+4 °C) accelerated the demise of freshwater diatom blooms caused by chytrid infections [25,26]. A marked predominance of *H. triquetra* over *A. minutum* was observed at temperatures below 18 °C (Figure 5A,B), with both dinoflagellates showing similar abundances at 20–22 °C (Figure 5E).

The results from the numerical simulations were consistent with the trends observed in the Penzé Estuary, where most *H. triquetra* and *A. minutum* blooms have been observed at 14–17 °C and 14–15 °C, respectively (Alves-de-Souza et al., in prep.). With a few exceptions, *H. triquetra* is the most predominant species in early summer at temperatures below 18 °C (when the cell concentration of this species frequently exceeds 1 × 10^6^ cells L^−1^; e.g., [9]) with *A. minutum* being more prevalent (but not at bloom levels) in the middle summer when temperatures surpass 19 °C [9]. Another congruence with our simulation outcomes was the increase in *P. rostrata* abundance, typically observed in the middle summer (positively correlated with temperature increases [9,27]) following periods of *A. minutum* predominance. Our experimental results and numerical simulations suggested that *H. triquetra* and *A. minutum* blooms in the Penzé Estuary could be related to a thermal refuge from *P. rostrata* infections due to low *P. rostrata* infectivity at low temperatures. In the case of *H. triquetra*, this advantage would be enhanced by its higher growth rates at 15–18 °C, resulting in much higher abundances when compared with *A. minutum*. Our results also offered an explanation for a perceived host preference by *Parvilucifera* parasitoids in field conditions [9,10] even when they presented a broader host range in the culture conditions [18,28,29]; these parasitoids potentially showed a preference for dinoflagellates occurring at temperature levels prone to their infections. Further studies are needed to assess if the *P. rostrata* responses to temperature gradients are also observed in other *Parvilucifera* species. One discrepancy between the model and field data in the Penzé Estuary is the absence of *A. minutum* and *H. triquetra* blooms in the late spring at temperatures around 13 °C when the field dinoflagellate assemblages are instead dominated by *Heterocapsa rotundata* [6]. A possible explanation could be a superior competitive advantage of *H. rotundata* at lower irradiance levels [30] observed in the late spring. The inclusion of the physiological responses to other environmental drivers other than temperature (e.g., irradiance and nutrient uptake) would allow for increasing the model predictability.

## 4. Concluding Remarks

This study represents the first comprehensive assessment of the effect of temperature on dinoflagellate infections by a unicellular eukaryote parasitoid. Our observations suggested that the nature of the temperature effect on the parasitoid functional response was host-dependent, varying from linear to unimodal in the same parasitoid infecting two different hosts. Although our experimental results and proposed modeling approach represent a simplification of the field conditions, they offer a valuable insight into the relative importance of temperature and its role in host–parasitoid dynamics and dinoflagellate succession in the field. Specifically, our results offer an explanation of the numerical mechanisms behind the thermal refuges of phytoplankton species to parasitoid infections at low temperatures observed in field studies. Further studies should consider mesocosm experiments to explore dinoflagellate–parasitoid interactions in the field, focusing on interactions with other physico-chemical drivers and biological interactions such as competition with sympatric parasitoids (e.g., *Amoebophrya* spp. in the Penzé Estuary) as well as potential allelopathic responses from dinoflagellate hosts. 

## Figures and Tables

**Table 1:** Values of the parameters and state variables considered in the numerical simulations.

State Variable/Parameters		Unit	Values				
**State variables**							
*K*	Carrying capacity	cells mL^−1^	40,000				
*H*_1_	*Alexandrium minutum*	cells mL^−1^	1000				
*H*_2_	*Heterocapsa triquetra*	cells mL^−1^	1000				
*P*	*Parvilucifera rostrata* zoospores	cells mL^−1^	2000				
*I*_1_	Infected *A. minutum*	cells mL^−1^	0				
*I*_2_	Infected *H. triquetra*	cells mL^−1^	0				
**Parameters**			**13 °C**	**15 °C**	**18 °C**	**20 °C**	**22 °C**
*r*_1_	*A. minutum* growth rate	d^−1^	0.16	0.23	0.30	0.37	0.31
*r*_2_	*H. triquetra* growth rate	d^−1^	0.50	0.53	0.56	0.48	0.31
*a*_1_	*P. rostrata* attack rate on *A. minutum* ^a^	d^−1^ × 10^3^	0.03	0.08	0.15	0.17	0.17
*a*_2_	*P. rostrata* attack rate on *H. triquetra* ^a^	d^−1^ × 10^3^	0.02	0.40	0.32	0.09	0.09
*h*	*P. rostrata* handling time	d	5	5	4	4	3
ε_1_	Zoospores per infected *A. minutum* ^b^	zoospores host^−1^	105	105	105	105	105
ε_2_	Zoospores per infected *H. triquetra* ^b^	zoospores host^−1^	7494	74	74	74	74
*m*	Zoospore mortality ^c^	d^−1^	3	3	3	3	3

^a^ Values estimated from Equations (4) and (5) were divided by 1000 to give per parasite rates; ^b^ Average values from data for each temperature; ^c^ Differences in zoospore mortality among hosts. Temperatures were not significant (see results); thus, for model purposes, we considered an average value from all data for the two species in all tested temperatures.

## Data Availability

The data presented in this study are available upon request to the corresponding author (C.A.-d.-S.).

## Supplementary Materials

The following supporting information can be downloaded at: https://www.mdpi.com/article/10.3390/microorganisms10020385/s1: Figure S1: Procedure for harvesting fresh *Parvilucifera zoospores*; Figure S2: Effect of temperature on the functional response of *Parvilucifera rostrata* RCC2800 (**A**,**B**) and RCC2823 (**C**,**D**) infecting the dinoflagellates *Alexandrium minutum* (left side) and *Heterocapsa triquetra* (right side); Figure S3: Numerical simulations of the effect of temperature on the dinoflagellate hosts *Alexandrium minutum* (**A**,**C**) and *Heterocapsa triquetra* (**B**,**D**) growing by themselves (right side) and together (left side) in the absence of the parasitoid; Figure S4: Results of simulations of *Parvilucifera rostrata* infections in *Alexandrium minimum* (**A**–**D**) and *Heterocapsa triquetra* (**E**–**H**) growing by themselves. Conceptual models used for numerical simulations are included as insets in (**A**,**E**).

## References

[1] Kagami M., Bruin A., Ibelings B.W., Donk E. (2007). Parasitic chytrids: Their effects on phytoplankton communities and foodweb dynamics. Hydrobiologia.

[2] Coats D.W., Park M.G. (2002). Parasitism of photosynthetic dinoflagellates by three strains of *Amoebophrya* (Dinophyta): Parasite survival, infectivity, generation time, and host specificity. J. Phycol..

[3] Montagnes D.J.S., Chambouvet A., Guillou L., Fenton A. (2008). Responsibility of microzooplankton and parasite pressure for the demise of toxic dinoflagellate blooms. Aquat. Microb. Ecol..

[4] Jephcott T.G., Alves-De-Souza C., Gleason F.H., van Ogtrop F.F., Sime-Ngando T., Karpov S.A., Guillou L. (2016). Ecological impacts of parasitic chytrids, syndiniales and perkinsids on populations of marine photosynthetic dinoflagellates. Fungal Ecol..

[5] Coats D.W., Bockstahler K.R. (1994). Occurrence of the parasitic dinoflagellate *Amoebophrya ceratii* in Chesapeake Bay populations of *Gymnodinium sanguineum*. J. Eukaryot. Microbiol..

[6] Chambouvet A., Morin P., Marie D., Guillou L. (2008). Control of toxic marine dinoflagellate blooms by serial parasitic killers. Science.

[7] Alacid E., Reñé A., Camp J., Garcés E. (2017). In situ occurrence, prevalence and dynamics of *Parvilucifera* parasitoids during recurrent blooms of the toxic dinoflagellate *Alexandrium minutum*. Front. Microbiol..

[8] Chambouvet A., Alves-de-Souza C., Cueff V., Marie D., Karpov S., Guillou L. (2011). Interplay between the parasite *Amoebophrya* sp. (Alveolata) and the cyst formation of the red tide dinoflagellate *Scrippsiella trochoidea*. Protist.

[9] Blanquart F., Valero M., Alves-de-Souza C., Dia A., Lepelletier F., Bigeard E., Jeanthon C., Destombe C., Guillou L. (2016). Evidence for parasite-mediated selection during short-lasting toxic algal blooms. Proc. R. Soc. B.

[10] Reñé A., Timoneda N., Sampedro N., Alacid E., Gallisai R., Gordi J., Fernández-Valero A.D., Pernice M.C., Flo E., Garcés E. (2021). Host preferences of coexisting Perkinsea parasitoids during coastal dinoflagellate blooms. Mol. Ecol..

[11] Alacid E., Reñé A., Garcés E. (2015). New insights into the parasitoid *Parvilucifera sinerae* life cycle: The development and kinetics of infection of a bloom-forming dinoflagellate host. Protist.

[12] Garcés E., Alacid E., René A., Petrou K., Simo R. (2013). Host-released dimethylsulphide activates the dinoflagellate parasitoid *Parvilucifera sinerae*. ISME J..

[13] Alacid E., Reñé A., Gallisai R., Paloheimo A., Garcés E., Kremp A. (2020). Description of two new coexisting parasitoids of blooming dinoflagellates in the Baltic sea: *Parvilucifera catillosa* sp. nov. and *Parvilucifera* sp. (Perkinsea, Alveolata). Harmful Algae.

[14] Llaveria G., Garcés E., Ross O.N., Figueroa R.I., Sampedro N., Berdalet E. (2010). Small-scale turbulence can reduce parasite infectivity to dinoflagellates. Mar. Ecol. Prog. Ser..

[15] Holling C.S. (1959). Some characteristics of simple types of predation and parasitism. Can. Entomol..

[16] Andersen R.A. (2005). Algal Culturing Techniques.

[17] Starr R., Zeikus J. (1993). UTEX-The culture collection of algae at the University of Texas at Austin. J. Phycol..

[18] Lepelletier F., Karpov S.A., le Panse S., Bigeard E., Skovgaard A., Jeanthon C., Guillou L. (2014). *Parvilucifera rostrata* sp. nov. (Perkinsozoa), a novel parasitoid that infects planktonic dinoflagellates. Protist.

[19] Ogle D.H., Doll J., Wheeler P., Dinno A. (2019). FSA: Simple Fisheries Stock Assessment Methods v. R Package Version. https://cran.r-project.org/web/packages/FSA/FSA.pdf.

[20] CRAN The Comprehensive R Archive Network. https://cran.r-project.org/.

[21] Guillard R., Stein J. (1973). Methods for microflagellates and nannoplankton. Handbook of Phycological Methods. Culture Methods and Growth Measurements.

[22] Salomon P.S., Stolte W. (2010). Predicting the population dynamics in *Amoebophrya* parasitoids and their dinoflagellate hosts using a mathematical model. Mar. Ecol. Prog. Ser..

[23] Arancio M. (2014). Etude Théorique des Interactions entre des Dinoflagellés et des Parasitoïdes Eucaryotes en Environnement Mélangé: Persistance du Système et Succession Phytoplanctonique/Theoretical study of Dinoflagellates and Eukaryot Parasitoids Interactions in Mixed Environment: System Persistence and Phytoplanktonic Succession. Ph.D. Thesis.

[24] Norén F., Moestrup Ø., Rehnstam-Holm A.S. (1999). *Parvilucifera infectans* Norén et Moestrup gen. et sp. nov. (Perkinsozoa phylum nov.): A parasitic flagellate capable of killing toxic microalgae. Eur. J. Protistol..

[25] Frenken T., Velthuis M., de Senerpont Domis L.N., Stephan S., Aben R., Kosten S., van Donk E., van de Waal D.B. (2016). Warming accelerates termination of a phytoplankton spring bloom by fungal parasites. Glob. Change Biol..

[26] Velthuis M., de Senerpont Domis L.N., Frenken T., Stephan S., Kazanjian G., Aben R., Hilt S., Kosten S., van Donk E., van de Waal D.B. (2017). Warming advances top-down control and reduces producer biomass in a freshwater plankton community. Ecosphere.

[27] Lepelletier F. (2013). Identification de Parasites Impliqués dans la Régulation des Efflorescences de la Microalgue Toxique *Alexandrium minutum*/Identification of Parasites Involved in the Bloom Control of the Toxic Microalgae *Alexandrium minutum*. Ph.D. Thesis.

[28] Figueroa R.I., Garcés E., Massana R., Camp J. (2008). Description, host-specificity, and strain selectivity of the dinoflagellate parasite *Parvilucifera sinerae* sp. nov. (Perkinsozoa). Protist.

[29] Garcés E., Alacid E., Bravo I., Fraga S., Figueroa R.I. (2013). *Parvilucifera sinerae* (Alveolata, Myzozoa) is a generalist parasitoid of dinoflagellates. Protist.

[30] Millette N., Pierson J., Aceves A., Stoecker D. (2017). Mixotrophy in *Heterocapsa rotundata*: A mechanism for dominating the winter phytoplankton. Limnol. Oceanogr..

