# Supplementary figures and images for: Temperature Affects the Biological Control of Dinoflagellates by the Generalist Parasitoid Parvilucifera rostrata
Source: Microorganisms. 2022 Feb 7;10(2):385. doi: 10.3390/microorganisms10020385 (PMC8874431; doi:10.3390/microorganisms10020385)

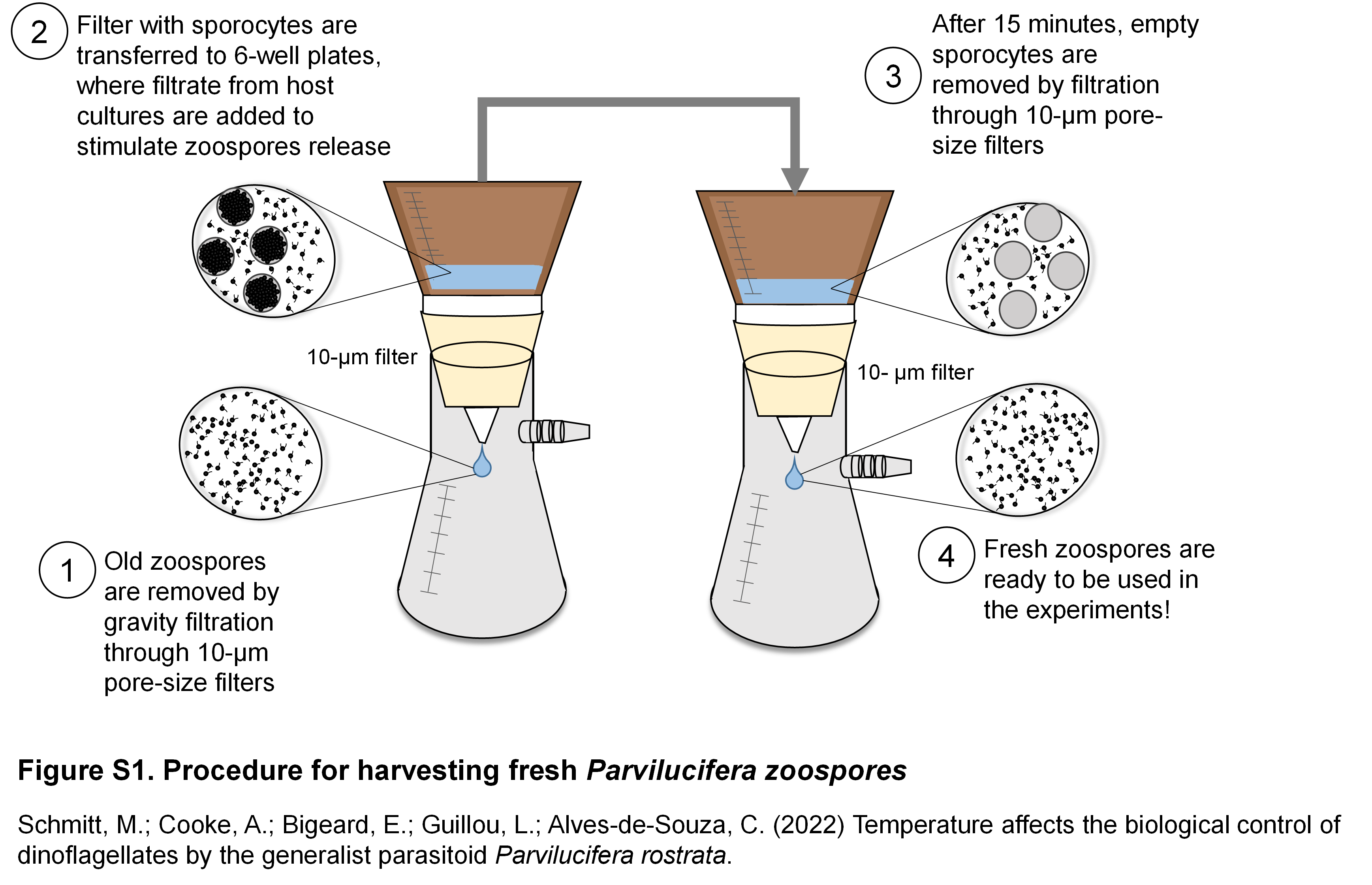

Supplement: Supplementary file 1 [file microorganisms-10-00385-s001.zip › Figure S1.png]

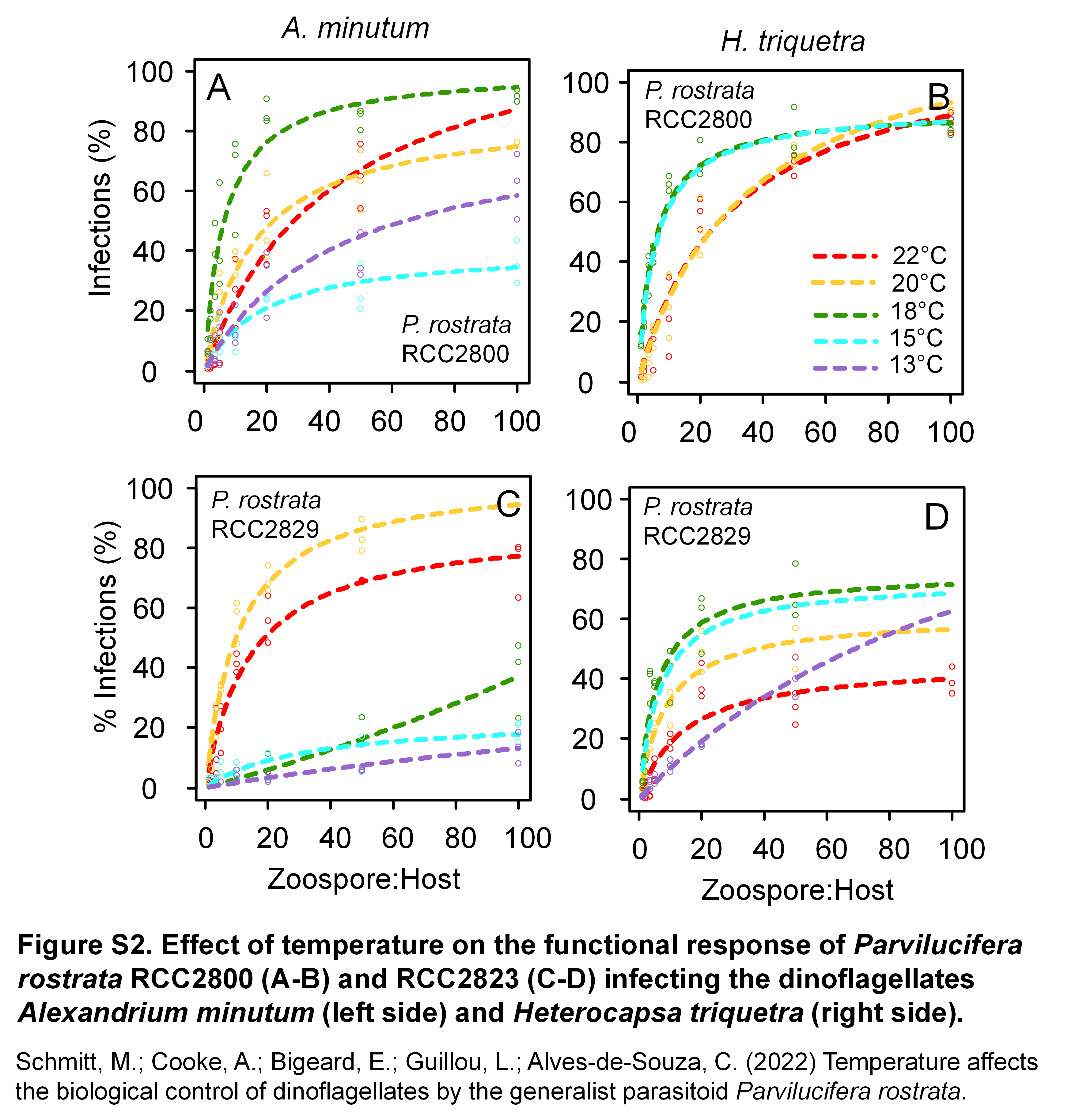

Supplement: Supplementary file 1 [file microorganisms-10-00385-s001.zip › Figure S2.png]

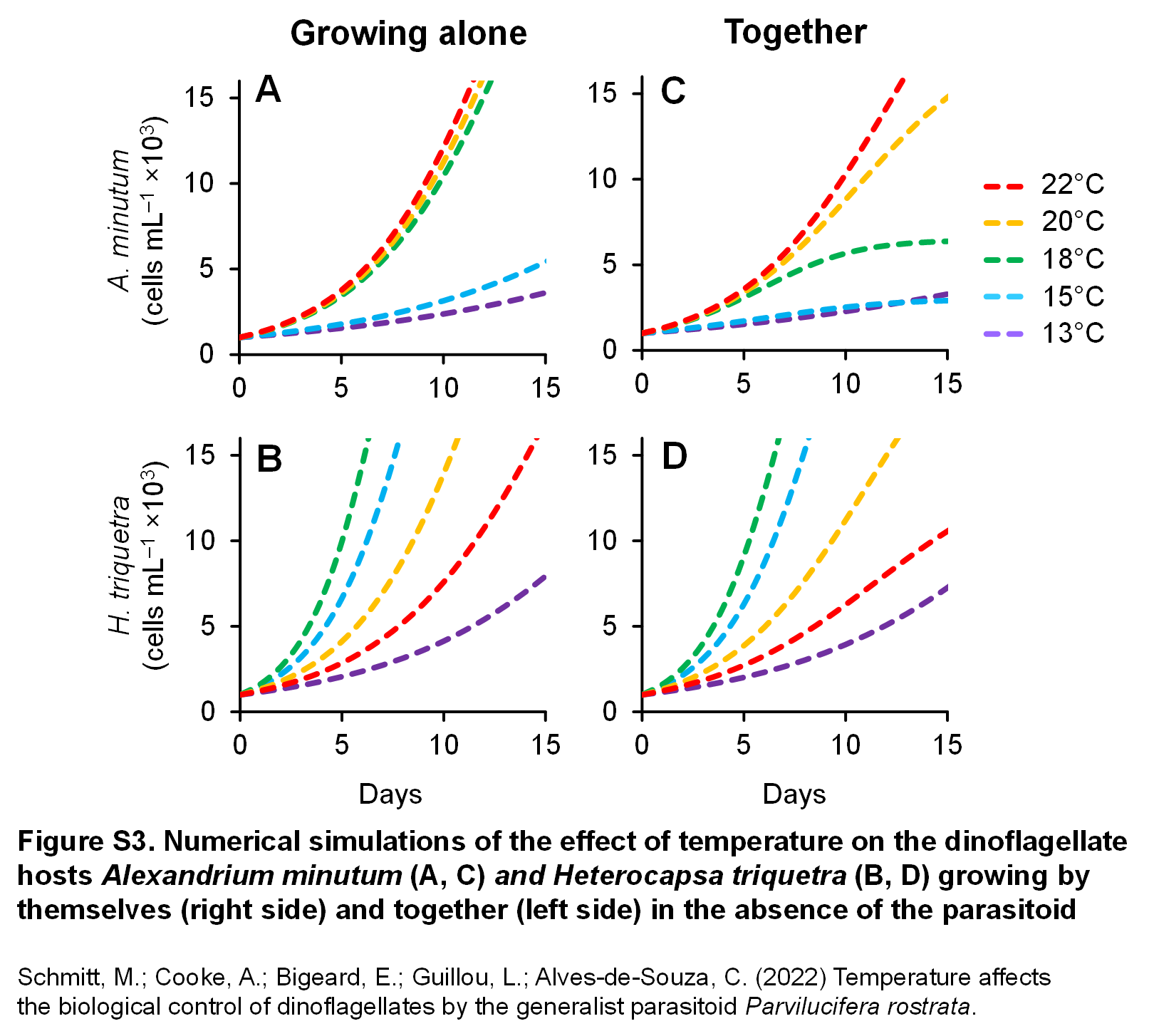

Supplement: Supplementary file 1 [file microorganisms-10-00385-s001.zip › Figure S3.png]

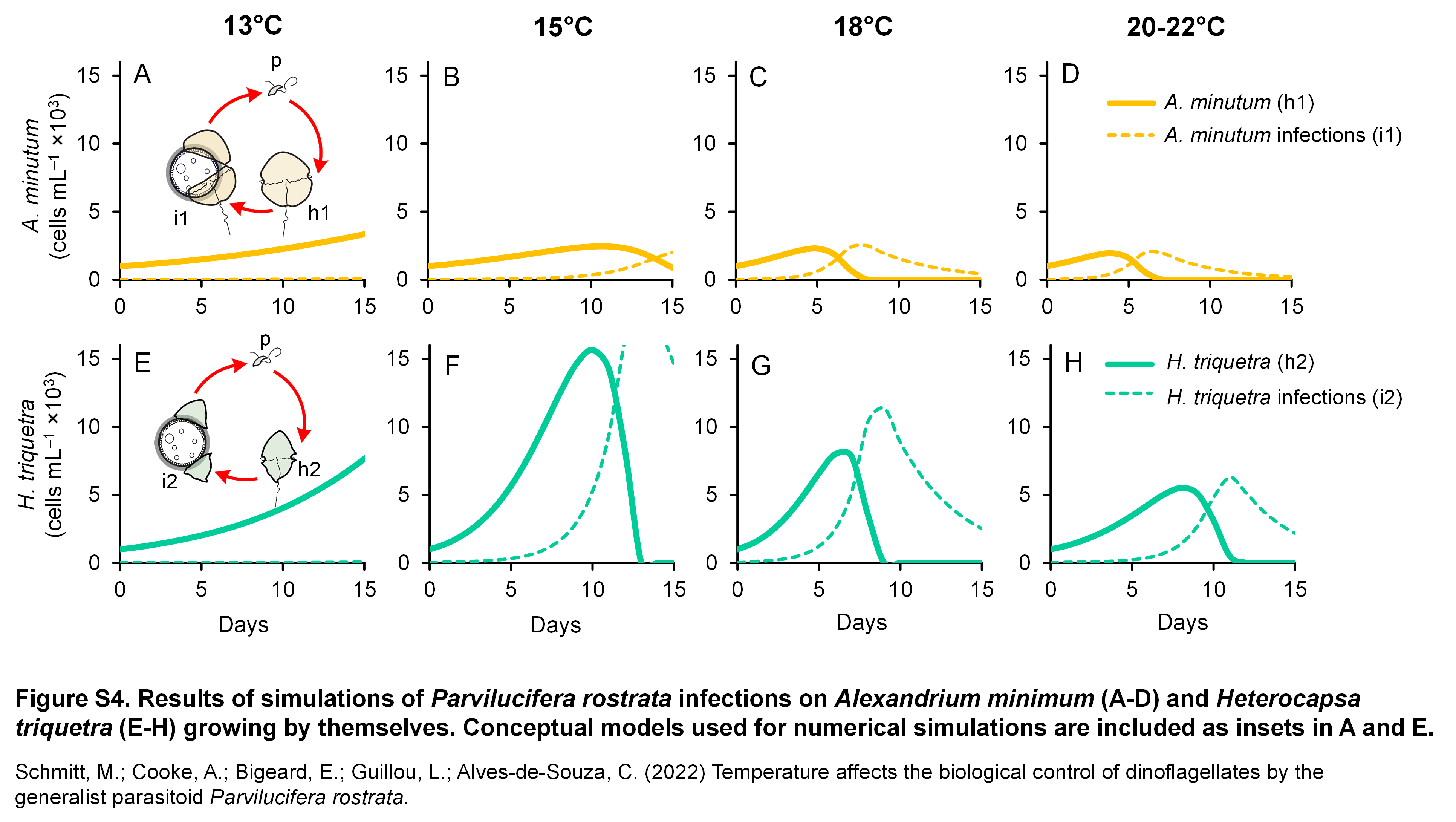

Supplement: Supplementary file 1 [file microorganisms-10-00385-s001.zip › Figure S4.png]
